# Fine-tuning *recA* expression in *Staphylococcus aureus* for antimicrobial photoinactivation: importance of photo-induced DNA damage in the photoinactivation mechanism

**DOI:** 10.1007/s00253-015-6863-z

**Published:** 2015-08-08

**Authors:** Mariusz Grinholc, Aleksandra Rodziewicz, Katarzyna Forys, Aleksandra Rapacka-Zdonczyk, Anna Kawiak, Anna Domachowska, Grzegorz Golunski, Christiane Wolz, Lili Mesak, Karsten Becker, Krzysztof P. Bielawski

**Affiliations:** Laboratory of Molecular Diagnostics, Department of Biotechnology, Intercollegiate Faculty of Biotechnology, University of Gdansk and Medical University of Gdansk, Kladki 24, 80-822 Gdansk, Poland; Department of Biotechnology, Division of Plant Protection and Biotechnology, Intercollegiate Faculty of Biotechnology, University of Gdansk and Medical University of Gdansk, Kladki 24, 80-822 Gdansk, Poland; Laboratory of Human Physiology, Medical University of Gdansk, Tuwima 15, 80-210 Gdansk, Poland; Laboratory of Biophysics, Intercollegiate Faculty of Biotechnology, University of Gdansk and Medical University of Gdansk, Kladki 24, 80-822 Gdansk, Poland; Interfaculty Institute of Microbiology and Infection Medicine, University of Tubingen, Wlfriede-Aulhorn-Strasse 6, 72076 Tubingen, Germany; Outreach, Research Training and Minority Science Program, Ayala School of Biological Sciences, University of California, 333 Steinhaus Hall, Irvine, CA 92697-2525 USA; Institute of Medical Microbiology, University Hospital Münster, Domagkstr. 10, 48149 Münster, Germany

**Keywords:** DNA damage, Endogenous porphyrins, Mutagenesis, Photoinactivation, RecA, *Staphylococcus aureus*

## Abstract

Bacterial cell envelope is generally accepted as the primary target for a photo-induced oxidative stress. It is plausible that DNA damage occurs during the antimicrobial photoinactivation. Here we investigate the correlation between DNA damage and photoinactivation by evaluating the level of RecA-based DNA repair system in *Staphylococcus aureus*. By using exogenous photosensitizers (new methylene blue (NMB), toluidine blue O (TBO), 5,10,15,20-tetrakis(1-methyl-4-pyridinio)porphyrin tetra(*p*-toluenesulfonate) (TMPyP), zinc phthalocyanine (ZnPc), Rose Bengal (RB)) and ALA-induced endogenous porphyrin-dependent blue light (405 nm), several outcomes were observed: (i) an increase of DNA damage (from gel electrophoresis in DNA damage assay), (ii) an increase of *recA* expression (luminescence assay in *recA*-*lux* strain), and (iii) an increase of RecA protein level (Western blotting). When *recA* expression was repressed by novobiocin, or abolished by deleting the gene, *S*. *aureus* susceptibility towards photoinactivation was increased at approximately a hundred-fold. The absence of RecA increases DNA damage to yield bactericidal effect. In novobiocin-resistant mutant (*gyrB*), as opposed to wild type, neither RecA protein level nor cell’s susceptibility was affected by photoinactivation (when novobiocin is present). This is to suggest that GyrB-dependent inhibition mediated *recA* repression. Therefore, we have established the role of RecA in DNA damage during photoinactivation. With the use of rifampicin mutation frequency and Ames tests, we demonstrated that photoinactivation did not increase *S*. *aureus* mutagenesis and potentially is not mutagenic toward eukaryotic cells. The results suggest that the treatment is considered safe. In conclusion, we provide an evidence that *recA* inhibitor may serve as therapeutic adjuvant for antimicrobial photoinactivation. Clinical relevance of our findings warrants further investigations.

## Introduction

Photoinactivation is one of the twentieth century’s antimicrobial discovery strategies against antibiotic-resistant pathogens, such as *Staphylococcus aureus*, by using three principle elements: photosensitizer (PS), visible light, and oxygen. In the presence of molecular oxygen and visible light, PS leads to the irreversible damage of various cell components (cell envelopes, lipids, proteins, and DNA) (Girotti 2001). With unspecific molecular targets, it is unlikely for bacteria to develop resistance against photodynamic processes. This approach is also called photodynamic inactivation (PDI), or antimicrobial photodynamic therapy (aPDT), and is being considered as a promising alternative for the treatment of localized infections (Yin et al. 2013). Several chemical classes of PSs that are evaluated against antimicrobial resistant bacteria upon illumination are (i) phenothiazines (new methylene blue, NMB; toluidine blue O, TBO), (ii) tetrapyrrolic macrocycles such as porphyrins (5,10,15,20-tetrakis(1-methyl-4-pyridinio)porphyrin tetra(*p*-toluenesulfonate), TMPyP), (iii) phthalocyanines (zinc phthalocyanine, ZnPc), (iv) xanthene dyes (Rose Bengal, RB), and (v) fullerenes (Kasimova et al. 2014; Quiroga et al. 2012; Mantareva et al. 2011; Kato et al. 2012; Grinholc et al. 2015). However, PDI can also be done using only blue light treatment with wavelengths of 405 or 470 nm in the presence of endogenous PS (Maclean et al. 2014; Rossi et al. 2012). PDI is a result of electron transfer, or energy transfer, from the light to its surroundings. During illumination, the PS in the ground state absorbs a photon and reaches an excited singlet state. Subsequently, during the course of losing energy, it returns to the ground state or can be converted into an excited triplet state. In this state, energy can be transferred to the surrounding substrates (type I) or to ground state molecular oxygen (type II), depending on the reaction type. Type I and type II reactions produce reactive oxygen species and excited singlet state oxygen, respectively. Both forms of reactive oxygen can cause two major cellular damages: the destruction of cell membranes and organelles and DNA damage (Foote 1991; Alves et al. 2014). The cell is protected by DNA repair systems; therefore, DNA damage may not be the main cause of cell death. The promising feature of PDI is to combat *S*. *aureus*, which may be improved by controlling DNA repair system, i.e., eliminating or inhibiting the expression of *recA*.

It is generally accepted that membrane proteins and other constituents of cell envelopes are major targets of photodynamic oxidation, even though some sensitizers can bind to DNA (Alves et al. 2014). Moreover, the photocleavage of bacterial DNA is considered to occur only when cells are largely photoinactivated or are no longer viable (Alves et al. 2014). If it was the case, the inhibition of the bacterial DNA repair system should not increase the photoinactivation efficacy. Current study shows that the bactericidal effectiveness of photoinactivation can be enhanced in the *recA*-negative strain or using an agent that downregulates *recA*, such as the aminocoumarin antibiotic agent novobiocin.

DNA damage triggers the SOS response, which is an inducible pathway for DNA repair. Two key proteins are involved in the SOS response: LexA (repressor) and RecA (inducer). In the absence of DNA damage, LexA binds to DNA to repress the transcription of several genes that are involved in DNA repair and cell division (16 genes in the case of *S*. *aureus*), including *lexA* and *recA* (Cirz et al. 2007). In response to DNA damage, the coprotease function of RecA is activated by binding to single-stranded DNA (ssDNA) and the formation of a nucleoprotein filament. RecA promotes the self-cleavage of LexA, leading to the derepression of SOS genes (Butala et al. 2009).

In previous reports, the *recA* expression was known to be activated by several classes of antibiotics, including fluoroquinolones, such as ciprofloxacin and inhibited by aminocoumarins (i.e., novobiocin) (Mesak et al. 2008; Schroder et al. 2013). In this study, the importance of DNA repair system in PDI was analyzed using (i) *S*. *aureus* HG001 *recA* and HG001 *lexAG94E* (Schroder et al. 2013), (ii) novobiocin-resistant *S*. *aureus* HG001 *nov142* (Schroder et al. 2014), and (iii) *S*. *aureus* promotor-*lux* constructs (*recA*-*lux* and *lexA*-*lux*) (Mesak et al. 2008). The use of these strains has supported the contribution of DNA damage to the efficacy of PDI.

In the present work, we explored whether DNA damage occurs during the PDI process and inhibition of the RecA-dependent DNA repair system could increase the treatment efficacy. We found out that photodynamic treatment elevated the expression of *recA* and RecA. Moreover, inactivation of *recA* reduced the viability of *S*. *aureus* following photodynamic treatment. Since photo-generated oxidative stress and the resultant DNA damage could induce bacterial mutagenesis (Kohanski et al. 2010), we also explored the mutagenic activity and impact of photodynamic treatment on *S*. *aureus* mutagenesis.

## Materials and methods

### Strains and growth conditions

The strains used in this study are listed in Table 1. The *S*. *aureus* strains were grown in brain-heart infusion broth (BHI media; BioMerieux, France). For the strains carrying resistance genes, antibiotics were used in overnight cultures at the following concentrations: erythromycin, ERY (10 mg/l; Sigma-Aldrich, Germany), and chloramphenicol, CHL (10 mg/l; Sigma-Aldrich, Germany). Bacteria from an overnight culture were diluted to an initial optical density (OD) at 600 nm (OD_600_) of 0.1 in fresh medium and grown with shaking (150 rpm) at 37 °C to the exponential growth phase (OD_600_ 0.6–0.7). Novobiocin (NOV) (Sigma-Aldrich, Germany) was added during the exponential growth phase at concentrations related to the minimum inhibitory concentration (MIC) of the strains (indicated in Table 1) and incubated for 1 h with shaking (150 rpm) at 37 °C. The MIC was determined as previously described (CLSI 2012).

**Table 1:** Strains used in this study

Strain	Description, MIC	Source
USA300 JE2	CA-MRSA USA300 JE2 strain, derived from USA300 LAC, NOV (0.06 mg/L), CIP (32 mg/L), RIF (0.06 mg/L)	NARSA
NE805	Mutant derived from USA300 JE2 strain deposited in Nebraska Transposon Mutant Library (NTML) with disrupted *recA* (recombinase A) gene by *bursa aurealis Tn* insertion, NOV (0.06 mg/L), CIP (32 mg/L)	NARSA
NCTC8325-4	Genome-sequenced laboratory strain, *rsbU* ^−^, NOV (0.06 mg/L)	(Novick 1967)
HG001	*rsbU* restored NCTC8325, NOV (0.5 mg/L)	(Herbert et al. 2010)
HG001 *recA*	HG001 with integrated pCG235 (*recA* PMUTIN), *recA* disrupted, cultured in the presence of ERY (10 mg/L), NOV (0.25 mg/L), CIP (0.06 mg/L)	(Schroder et al. 2013)
HG001 *lexAG94E*	HG001 with G94E substitution in the LexA, uncleavable LexA, NOV (0.5 mg/L)	(Schroder et al. 2013)
HG001 *nov142*	HG001 nov (*gyrB142*), NOV resistant derivative of HG001	(Schroder et al. 2014)
RN4220 *recA*-*lux*	The *recA* promoter-*lux* fusion construct of the restriction-deficient derivative of 8325-4 (RN4220), cultured in the presence of CHL (10 mg/L)	(Mesak et al. 2008)
RN4220 *lexA*-*lux*	The *lexA* promoter-*lux* fusion construct of the restriction-deficient derivative of 8325-4 (RN4220), cultured in the presence of CHL (10 mg/L)	(Mesak et al. 2008)
8325-4 *hemB*	Isogenic strain derived from 8325-4, *hemB* knockout mutant displaying the small colony variant (SCV) phenotype	(Von et al. 1997)

*CHL* chloramphenicol, *NOV* novobiocin, *CIP* ciprofloxacin, *RIF* rifampicin

### Photosensitizers

The following PSs were purchased from Sigma-Aldrich (Germany): new methylene blue (NMB), toluidine blue O (TBO), 5,10,15,20-tetrakis(1-methyl-4-pyridinio)porphyrin tetra(*p*-toluenesulfonate) (TMPyP), zinc phthalocyanine (ZnPc), and Rose Bengal (RB). Most of the PSs, excluding ZnPc, were dissolved in distilled water to produce 1 or 0.1 mM stock solutions and stored in the dark at −20 °C (1 mM for NMB, TBO, and TMPyP and 0.1 mM for RB). ZnPc was dissolved in dimethyl sulfoxide (DMSO) to obtain a 0.1 mM stock solution. The cationic C_60_ fullerene derivative (fulleropyrrolidine, FUL) was purchased from ProChimia (Poland). The compound was maintained in the dark at a concentration of 0.1 mM in dimethyl sulfoxide:ddH_2_O solution (*v*/*v*, 1:9) (Grinholc et al. 2015).

### Light sources

Illumination was performed using the following light sources: Q.Light® PDT Lamp (b & p® Schweiz AG, Switzerland) (ISO 9001 & EN 46001 - CE 1275) (lamp power of 80 mW). The Q.Light® PDT Lamp emits polarized light (polarization level 98 %) over a wavelength ranging from 620 to 780 nm (red filter, irradiance 102 mW/cm^2^), 385–480 nm (blue filter, irradiance 127 mW/cm^2^), and white light (no filter used, irradiance 267 mW/cm^2^). The LED illuminators had wavelengths of 627 nm (light wavelength 627 nm, power 50 W, irradiance set at 11 mW/cm^2^) and 405 nm (light wavelength 405 nm, power 120 W, irradiance set at 5 mW/cm^2^) and were custom designed for laboratory use (SecureMedia, Poland). The delivered light energy was determined with the use of a light power meter (model LM2, CARL ZEISS, Germany).

### Photoinactivation

Microbial overnight cultures were diluted with fresh medium to an optical density OD_600_ of 0.1 and cultured to exponential growth phase (OD_600_ 0.6–0.7) with shaking (150 rpm) at 37 °C. In the novobiocin-treated samples, the cultures were incubated for an additional 1 h with MIC concentration of novobiocin (150 rpm, 37 °C). The cultures were centrifuged (1000*g*) for 3 min, washed twice with PBS, and resuspended in fresh BHI medium, and the concentration was adjusted to 10^7^ CFU/ml. The bacterial suspensions were administered with PSs and incubated for 30 min at room temperature in the dark (all PSs and their concentrations used in this study are listed in Table 2). A 100 μl aliquot of the cell suspension were transferred to a 96-well plate. The highest concentration of DMSO used was below 0.1 % and was not toxic to the cells. Cells were then illuminated with the appropriate light dose (characteristic of the light sources and applied light doses are listed in Table 2). At the completion of the illumination period, 10-μl aliquots were removed from the illuminated and non-illuminated wells (control cells were maintained in 96-well plates covered with aluminum foil at room temperature for the duration of the illumination) and serially diluted tenfold in PBS to generate dilutions of 10^−1^ to 10^−4^ times the original concentrations. Afterward, 10-μl aliquots of each dilution were streaked horizontally on square Petri dishes containing brain-heart infusion medium (BHI; BioMerieux, France) (Jett et al. 1997). The plates were streaked in triplicate and incubated for 24 h at 37 °C in the dark to allow colony formation. Controls groups included cells that were not treated with PSs or light and cells that were treated with light but not with PSs. In addition, control samples consisted of cultures that were treated with novobiocin but kept in the dark. The survival fractions (SF) are expressed as ratios of the CFU of microbial cells treated with photoinactivation to the CFU of non-treated microbes. Each experiment was performed three times for statistical analysis.

**Table 2:** Sublethal (<2 log_10_ unit reduction in viable counts) doses of phototreatment

Sensitizer, concentration (μM)	Light source	Log_10_ reduction
NMB, 50	LED illuminators 627 nm, irradiance 11 mW/cm^2^, fluence 50 J/cm^2^	0.45
TBO, 100	LED illuminators 627 nm, irradiance 11 mW/cm^2^, fluence 50 J/cm^2^	0.7
TMPyP, 100	LED illuminators 627 nm, irradiance 11 mW/cm^2^, fluence 50 J/cm^2^	0.5
ZnPc, 5	Q.Light® PDT Lamp 620–780 nm (red filter), irradiance 102 mW/cm^2^, fluence 100 J/cm^2^	0.6
RB, 5	Q.Light® PDT Lamp 385–480 nm (blue filter), irradiance 127 mW/cm^2^, fluence 200 J/cm^2^	1.0
FUL, 1	Q.Light® PDT Lamp white light (no filter used), irradiance 267 mW/cm^2^, fluence 80 J/cm^2^	0.9
None	LED illuminators 405 nm, irradiance 5 mW/cm^2^, fluence 10 J/cm^2^	0.1 (USA300 JE2), 0.5 (HG001), 0.1 (8325-4)

*NMB* new methylene blue, *TBO* toluidine blue O, *TMPyP* 5,10,15,20-tetrakis(1-methyl-4-pyridinio)porphyrin tetra(*p*-toluenesulfonate), *ZnPc* zinc phthalocyanine, *RB* Rose Bengal, *FUL* fulleropyrrolidine

### Photo-induced DNA damage assay

The cell suspension of *S*. *aureus* in mid-log growth phase in PBS was treated photodynamically as indicated in Table 2. Post-irradiation, the genomic DNA (10 ng) was extracted from the *S*. *aureus* cells using the Extract*ME* DNA Bacteria purification kit (Blirt S.A., Poland) and treated with Endonuclease III (BioLabs, New England). This treatment allows the visualization of the photo-induced damage in the genomic DNA. The genomic DNA samples were analyzed by electrophoresis (0.8 % agarose gel, with ethidium bromide staining at 120 V). Band intensities were measured as a percentage of the control sample using ImageJ 1.48v (Wayne Rasband, National Institutes of Health, USA; http://imagej.nih.gov/ij).

### Western blot analysis

The *S*. *aureus* strains were grown to an OD_600_ of 0.6 and challenged with photoinactivation and/or antibiotics (NOV and CIP) for 1 h (Tables 1 and 2). Three milliliters of the bacterial suspensions were harvested, washed with 5 ml Tris/EDTA (TE; 10 mM Tris, 1 mM EDTA, pH 7.4) and resuspended in 250 μl TE with protease inhibitor cocktail as recommended by the manufacturer (Roche, Germany). After incubation on ice for 10 min, the cells were lysed with 0.25 ml of zirconia beads (0.1 mm in diameter) in a high-speed homogenizer (Roche MagNA Lyser) twice for 20 s each at a speed of 6500 rpm. After centrifugation for 10 min at 4700*g*, the supernatant was transferred and again centrifuged for 10 min at 13,000*g*. The protein concentrations were determined in the bacterial cell lysates using the Bio-Rad Protein Assay Dye Reagent (Bio-Rad, Germany). Equal protein concentrations (10 μg) were subjected to SDS-PAGE, and the separated proteins were transferred to a nitrocellulose blotting membrane (GE Healthcare, Life Sciences, Germany) using a Bio-Rad Mini Trans-Blot cell. The membrane was blocked at room temperature for 1 h with 5 % non-fat dry milk in PBS, pH 7.5. For immunostaining, polyclonal rabbit RecA antibodies (Abnova, Taiwan; 1:1000) were used for an overnight incubation (4 °C). This was followed by a 1-h incubation with secondary horseradish peroxidase (HRP)-conjugated anti-rabbit IgG antibodies (Cell Signaling, Germany; 1:2000) and detection of proteins by enhanced chemiluminescence (ChemiDoc, Bio-Rad) with a HRP substrate (SuperSignal, Thermo Scientific, Germany).

### Cell membrane integrity

For the cell membrane studies, *S*. *aureus* in mid-log growth phase was centrifuged (1000*g*, 3 min), washed, and resuspended in PBS, and then it was exposed to 405-nm irradiation or control conditions (maintained in the dark). Additionally, untreated bacteria were exposed to 0.01 % benzethonium chloride (BCl; cell membrane disruption, positive control) for 1 h. Then, propidium iodide (PI; at a final concentration of 5 μg/ml) was placed in each sample, and the cells were incubated for an additional 30 min in the dark at room temperature. Following staining, the bacteria were centrifuged, washed, and resuspended in 1-ml PBS. The fluorescence of the samples was read using an EnVision Multilabel Plate Reader (PerkinElmer) with 488/570 nm excitation and emission filters. Additionally, the samples were stained with SYTOX Green (Molecular Probes) at a final concentration of 5 μM for 10 min at room temperature. Finally, the fluorescence signal of DNA-bound SYTOX Green was measured using an EnVision Multilabel Plate Reader (Perkin Elmer) with 504/523 nm excitation and emission filters. The experiments were performed three times and analyzed statistically (Connell et al. 2013).

### Rifampicin mutation frequency determinations

The mutation frequencies were determined as previously described (O’Neill et al. 2001). Bacterial suspensions in mid-log phase, after photodynamic treatment, were spread onto selective BHI plates containing 4× MIC of rifampicin to recover spontaneous antibiotic-resistant mutants. The culture dilutions were also spread onto non-selective BHI to determine the viable cell numbers. The agar plates were incubated for 24–48 h at 37 °C, and the mutation frequencies are expressed as the number of antibiotic-resistant mutants recovered as a proportion of the total cell count. The mutation frequencies were determined for three biological replicates.

### Ames mutagenicity assay

The histidine-dependent (His^−^) *Salmonella enterica* serovar Typhimurium (*S*. Typhimurium) TA98 strain and pooled S9 enzymatic fraction from rat liver induced with Aroclor (stored at −80 °C) were purchased from Xenometrics (Stilwell, USA). The S9 fraction was mixed with cofactors at a volume ratio of 1:24. The cofactors for the S9 mixture contained 19 mM MgCl_2_, 36 mM KCl, 4.5 mM NADP, and 5.2 mM d-glucose-6-phosphate in 0.1 M sodium phosphate buffer (pH 7.4). The *Salmonella* mutagenicity assay (Ames test) was conducted with preincubation as described previously (Mortelmans and Zeiger 2000) with modifications. For the metabolic activation assay, 50 μl of the overnight culture (cultivated for 4 h at room temperature and then for 12 h at 37 °C with shaking) were incubated with 133 μl of S9 mix and 50 μl of the tested chemical for 20 min at 37 °C. Next, 67 μl of solution containing 0.1 μmol of histidine and 0.1 μmol of biotin was added to the mixture, which was then plated on the agar plate with minimal glucose medium, as previously described (Woziwodzka et al. 2013). For the assays conducted without metabolic activation, a previously reported procedure was used (Golunski et al. 2013; Woziwodzka et al. 2011). A mixture containing 50 μl of the overnight culture (cultivated for 4 h at room temperature and then for 12 h at 37 °C with shaking), 60 μl of 3 % NaCl, and 150 μl of the tested chemical was incubated with shaking for 4 h at 37 °C. After incubation, the mixture was centrifuged, and the bacteria were washed and resuspended in 300 μl of 0.6 % NaCl plating solution containing 0.1 μmol of histidine and 0.1 μmol of biotin. Finally, the bacteria were plated on an agar plate containing minimal glucose medium. The plates were incubated for 48 h at 37 °C in the dark, and then the His^+^ revertants were counted. All of the experiments were performed in triplicate. The cytotoxic effects on the bacteria were determined by observing the background lawn. For the negative control, the tested chemical was replaced with distilled water. All of the results are presented as a percentage of the mutagenic activity of the positive control. The results of Ames test were evaluated statistically using Statistica 9.1 (StatSoft) software. One-way analysis of variance (ANOVA) followed by the post-hoc RIR Tukey’s test was applied to evaluate the results. The significance level was established at *α* = 0.05.

### Luminescence measurements

Liquid assays were performed at room temperature in a clear-bottom 96-well plate by using starting cultures with an optical density at 595 nm of 0.15. The bacteria were treated photodynamically or with ciprofloxacin, and the luminescence was recorded every 2 h for 6 h in a Wallac 1420 Victor multilabel counter (Perkin-Elmer). Each determination was replicated three times.

### Porphyrin extraction and fluorescence measurements

It was possible to detect the produced porphyrins based on their extraction with MeOH:acetone (1:1, *v*/*v*). Extracellular porphyrins, which were excreted from the cells, were extracted from the growth medium supernatant after lyophilization (Thermo Electron Corporation, Heto PowerDry LL3000 Freeze Dryer) and sonication (Polsonic, Sonic-5) (10 min). After extraction of the porphyrins, their fluorescence spectra were determined using a Perkin Elmer Multimode Plate Reader EnVision® spectrofluorometer. The excitation wavelength was 405 nm. To induce endogenous porphyrin production, delta-aminolevulinic acid (1 mM) was added to the bacterial suspensions and cultured for 48 h at 37 °C with shaking (150 rpm).

### Statistical methods

The means of the survival and bioluminescence fractions were analyzed by one-way ANOVA. *p* values less than 0.05 were considered significant.

## Results

### Phototreatment leads to DNA damage

**Fig. 1:**
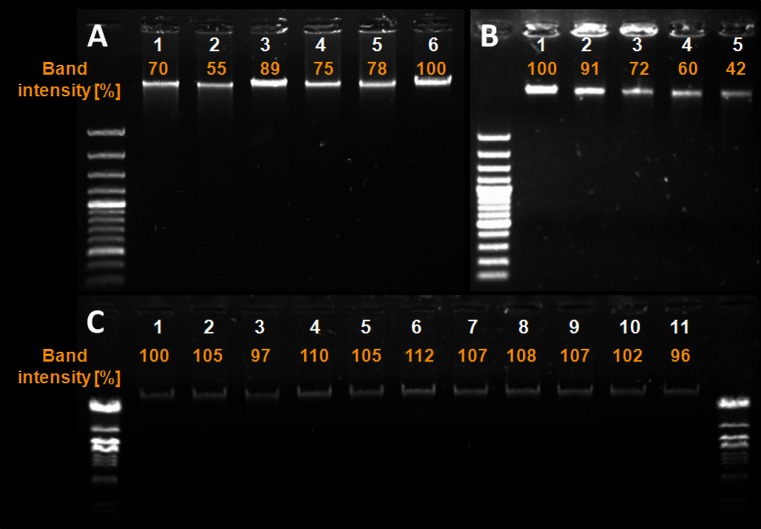
Agarose gel electrophoresis showing the genomic DNA of untreated and treated samples. **a** Genomic DNA damage of *S*. *aureus* USA300 JE2 treated photodynamically according to Table 2 with NMB (*lane 1*), TBO (*lane 2*), TMPyP (*lane 3*), ZnPc (*lane 4*), RB (*lane 5*), and FUL (*lane 6*). Band intensity measured as a percentage of the control (bacteria treated with FUL). **b** Genomic DNA damage of *S*. *aureus* USA300 JE2 treated with light at 405 nm: *lane 1*: non-treatment control (bacteria kept in dark); *lanes 2*–*5*: phototreated bacteria (10, 20, 30, and 40 J/cm^2^). Band intensity measured as a percentage of the control (bacteria kept in dark). **c** Genomic DNA damage of *S*. *aureus* USA300 JE2 treated with control conditions: *lane 1*: bacteria kept in the dark; *lanes 2*–*7*: bacteria treated with sensitizers in the dark (NMB, TBO, TMPyP, ZnPc, RB, and FUL, respectively); *lanes 8*–*11*: bacteria treated with light (627 nm, irradiance 11 mW/cm^2^, fluence 50 J/cm^2^; 620–780 nm (red filter), irradiance 102 mW/cm^2^, fluence 100 J/cm^2^; 385–480 nm (blue filter), irradiance 127 mW/cm^2^, fluence 200 J/cm^2^; white light (no filter used), irradiance 267 mW/cm^2^, fluence 80 J/cm^2^). Band intensity measured as a percentage of the control (bacteria kept in dark)

**Fig. 2:**
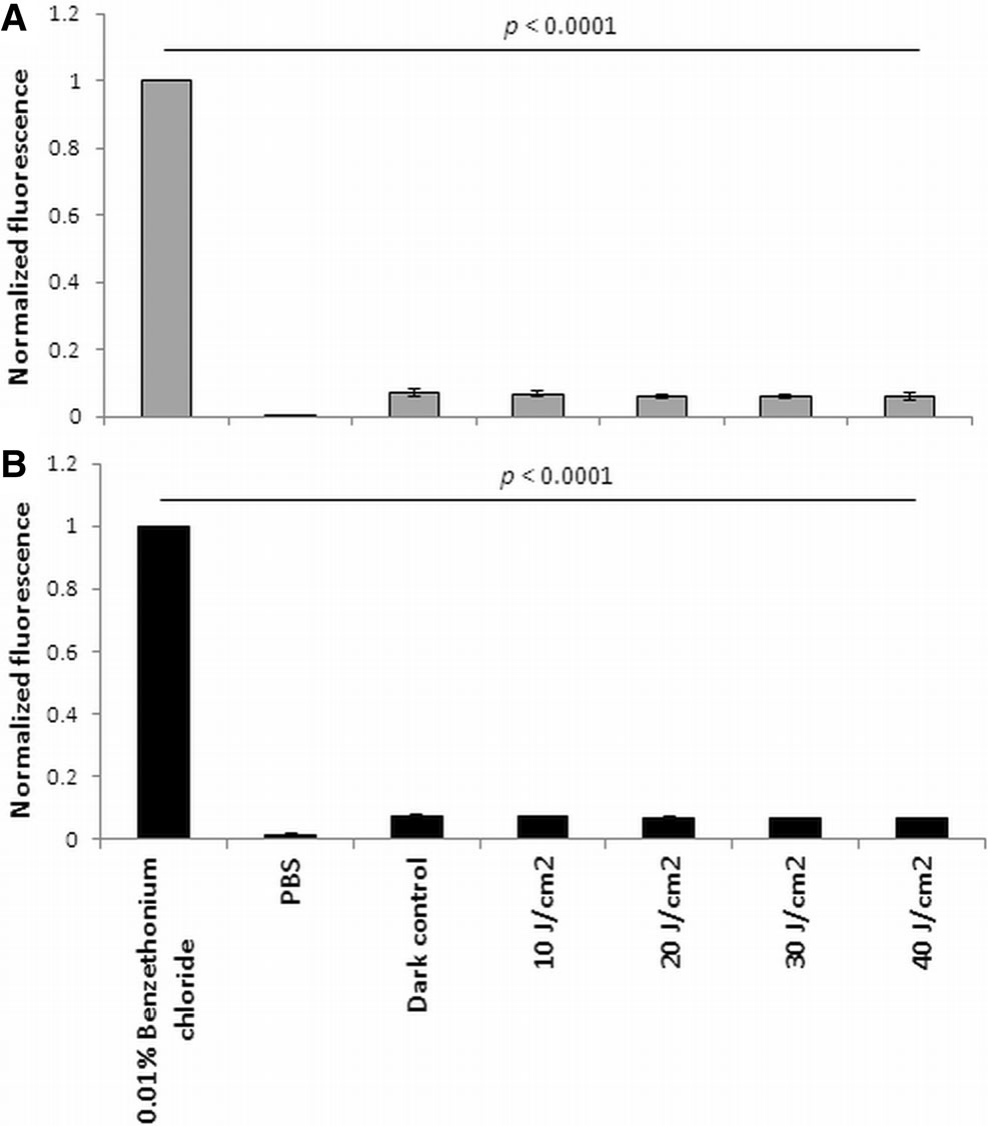
*S*. *aureus* cell membrane integrity. **a** Total SYTOX Green fluorescence readings for different groups. **b** Total propidium iodide fluorescence readings for different groups. All of the groups were statistically significant compared with the negative and positive control. All of the values were normalized with respect to the same bacterial populations treated for 1 h with BCl (0.01 %). Significance of comparisons between treated samples and control, *p* < 0.0001

Photoinactivation induce DNA damage via the direct interaction between excited sensitizer molecules with DNA, by reactive oxygen species-mediated reactions, or reactions involving other secondary intermediates (Epe 2012). However, it is accepted that this effect might only be observed in largely photo-induced bacterial cells. Thus, we investigated whether DNA damage could be detected using sublethal photoinactivation (resulting in <2 log_10_ unit reduction). Upon administration of the sensitizer and sample irradiation, the *S*. *aureus* USA300 JE2 genomic DNA was extracted, treated with endonuclease III, and resolved by agarose gel electrophoresis (Fig. 1). As shown in Fig. 1a, excluding fulleropyrrolidine-mediated treatment, exogenous administration of the sensitizer upon irradiation resulted in significant DNA damage indicated by the presence of DNA smear. Additionally, the DNA damage was semi-quantified using ImageJ software and presented as band intensities according to the control samples. In the case of PDI-treated samples, the control consisted of *S*. *aureus* cultures treated with fulleropyrrolidine as previous studies indicated that this sensitizer do not lead to DNA damage (Grinholc et al. 2015). The same effect could also be observed with light treatment at 405 nm involving endogenously produced sensitizers (Fig. 1b). However, no DNA damage was reported in control samples consisting of *S*. *aureus* cultures treated with white, red, and wide range blue light or with sensitizers without light activation (Fig. 1c). The occurrence of DNA damage following phototreatment within viable cells was also confirmed using cell membrane integrity measurements. Blue light (405 nm) doses up to 40 J/cm^2^ resulted in a large amount of DNA damage (Fig. 1), but simultaneously, no disruption of the cell membrane integrity was detected in the phototreated cells (Fig. 2), which indicated that the majority of the bacterial population was still viable.

### RecA contributes to the phototreatment outcome

**Fig. 3:**
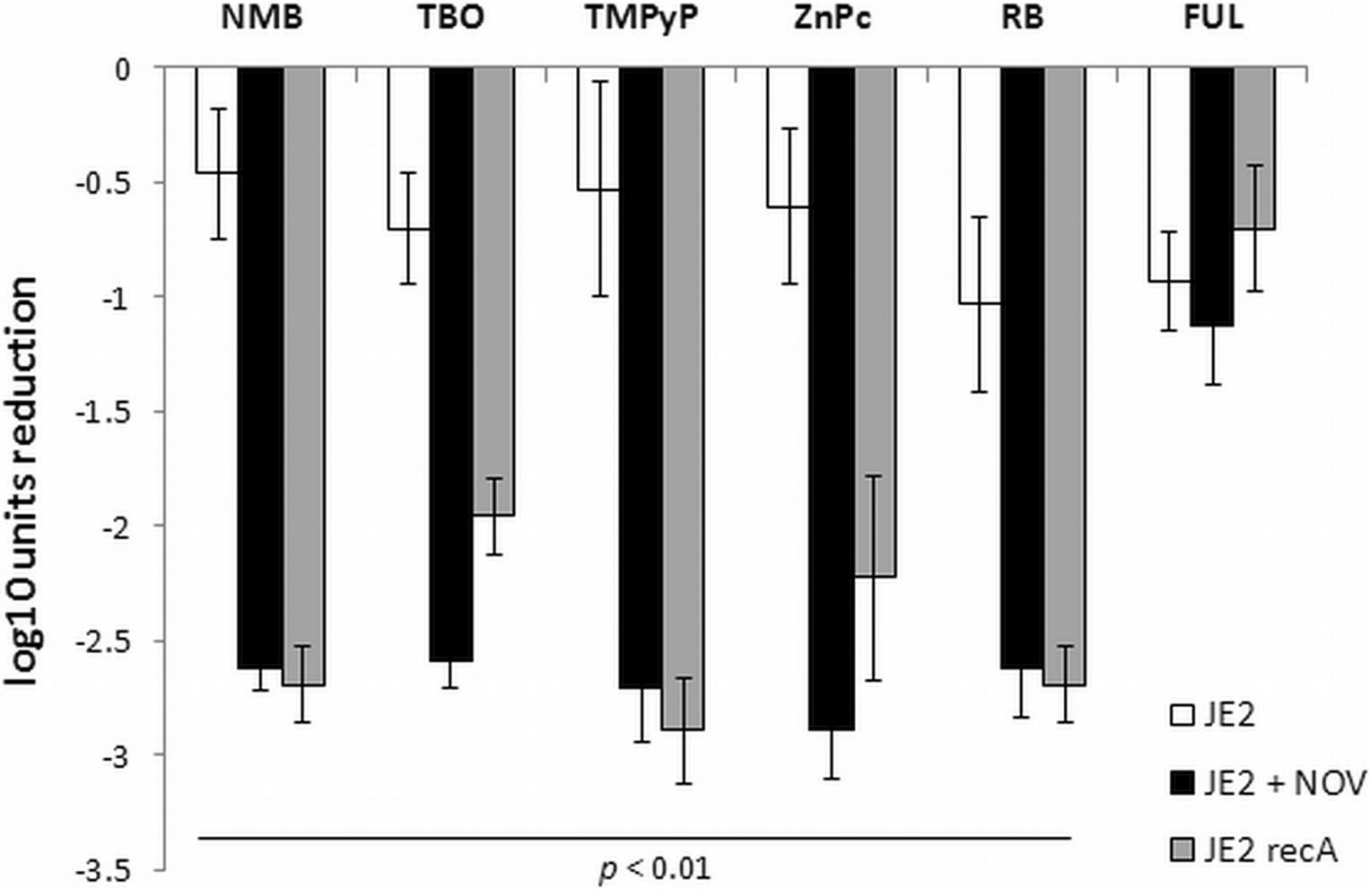
Photoinactivation efficacy against *S*. *aureus* USA300 JE2. Microbial cell planktonic suspensions of *S*. *aureus* USA300 JE2 (*white bars*) and JE2 *recA* (*gray bars*) in mid-log phase were incubated for 30 min with various sensitizers and then illuminated in accordance with the conditions presented in Table 2. For the novobiocin-mediated experiments (*black bars*), mid-log phase cultures were preincubated with antibiotic (MIC) for 1 h at 37 °C. The values are means of three separate experiments, and the *bars* represent S.D. Significance of comparisons between novobiocin-treated samples and control, *p* < 0.01. Significance of comparisons between JE2 *recA* and wild-type strain, *p* < 0.01. *NMB* new methylene blue, *TBO* toluidine blue O, *TMPyP* 5,10,15,20-tetrakis(1-methyl-4-pyridinio)porphyrin tetra(*p*-toluenesulfonate), *ZnPc* zinc phthalocyanine, *RB* Rose Bengal, *FUL* fulleropyrrolidine, *NOV*, novobiocin

**Fig. 4:**
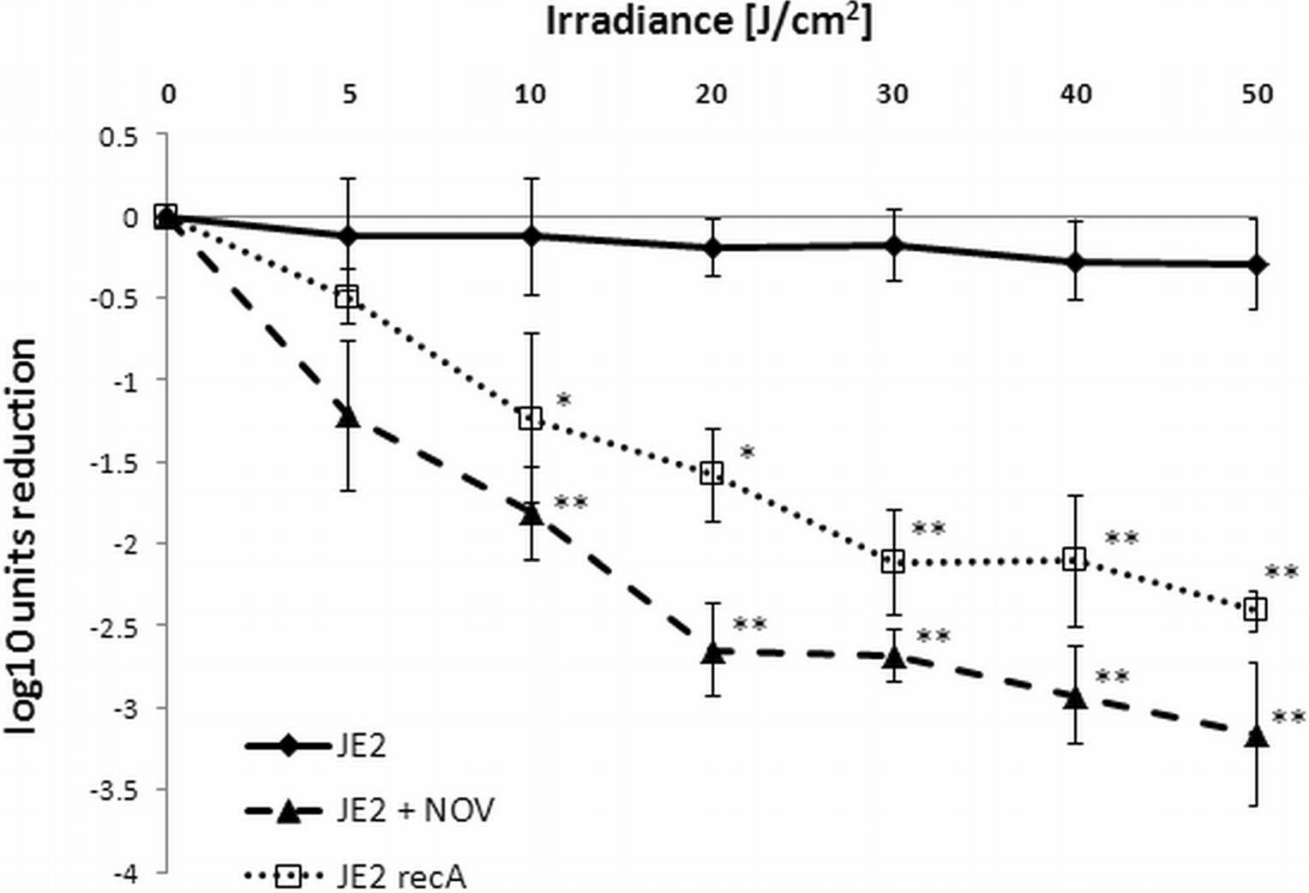
Blue light (405 nm) irradiation efficacy. Microbial cell planktonic suspensions of *S*. *aureus* USA300 JE2 (*black diamonds*) and JE2 *recA* (*open squares*) in mid-log phase were illuminated with blue light (405 nm) within a range of irradiances (0–50 J/cm^2^). For the novobiocin-mediated experiments (*black triangles*), mid-log phase cultures were preincubated with antibiotic (MIC) for 1 h at 37 °C. The values represent the means of three separate experiments, and the *bars* are the S.D. Significance of comparisons to the control, *p < 0.05, **p < 0.01. *NOV* novobiocin

Since DNA damage could be observed in response to phototreatment, one could assume that it promotes DNA repair mechanisms and leads to the induction of the SOS response, resulting in RecA activation. Thus, we investigated whether the presence of RecA or inhibition of the *recA* gene could influence the efficacy of the phototreatment. The wild-type USA300 JE2 and its isogenic daughter isolate JE2 *recA* (*recA*-negative, NE805) were treated by sublethal photoinactivation (Fig. 3). In addition, the wild-type strain was photoinactivated in the presence of MIC of novobiocin (*recA*-downregulating agent) (Fig. 3). The bactericidal effectiveness against these *S*. *aureus* strains was also analyzed using the 405-nm light treatment (Fig. 4). As indicated, the strain with disrupted (JE2 *recA*) or inhibited (novobiocin) *recA* expression demonstrated increased susceptibility to PDI, excluding the fulleropyrrolidine treatment. This result suggests that DNA damage occurs in phototreated cells and that the damage can be counteracted by RecA activity. Thus, the inhibition of *recA* expression as part of DNA repair mechanisms/SOS response system could increase the bactericidal efficacy of the PDI.

### The effect of novobiocin on the phototreatment efficacy is due to *recA* repression that mediated by GyrB-dependent inhibition

**Fig. 5:**
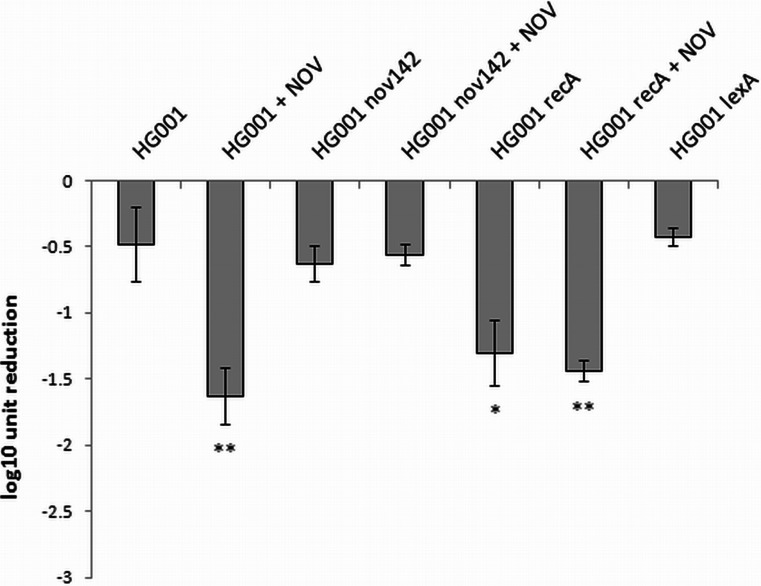
Photoinactivation efficacy against *S*. *aureus* HG001. Microbial cell planktonic suspensions of *S*. *aureus* HG001, HG001 *nov142*, HG001 *recA*, and HG001 *lexA* in mid-log phase were illuminated (405 nm, irradiance 5 mW/cm^2^, fluence 10 J/cm^2^). For the novobiocin-mediated experiments, mid-log phase cultures were preincubated with antibiotic (MIC) for 1 h at 37 °C. The means of three separate experiments are presented, and the *bars* represent the S.D. Significance of comparisons to the control, *p < 0.05, **p < 0.01. *NOV* novobiocin

We have previously shown that novobiocin downregulates the transcription of *recA* (Schroder et al. 2013; Mesak et al. 2008), and this effect is mediated by inhibition of the GyrB subunit (Schroder et al. 2014). To verify that the presence of novobiocin did not influence the phototreatment outcome via the GyrB-independent pathway, the bactericidal effectiveness of the photoinactivation was analyzed for the wild-type *S*. *aureus* strain (HG001) and its isogenic mutant strain expressing a novobiocin non-susceptible GyrB enzyme (HG001 *nov142*). This latter strain is resistant to novobiocin. To simplify the analysis, the indicated strains were treated only with the 405-nm light, and it was assumed that a similar effect would be observed for exogenously administered sensitizers. This assumption was warranted by the similar DNA damage upon photoinactivation and its *recA*-dependent efficacy. The parental strain revealed increased susceptibility to photodynamic treatment in the presence of novobiocin (Fig. 5). In the novobiocin-resistant strain, the effectiveness of 405-nm irradiation was comparable to that observed in the parental strain and was not affected by the presence of novobiocin. The results clearly indicate that the effect of novobiocin on the phototreatment efficacy is mediated by GyrB and is unlikely to be due to additional effects on other potential targets.

### Phototreatment increases *recA* expression

**Fig. 6:**
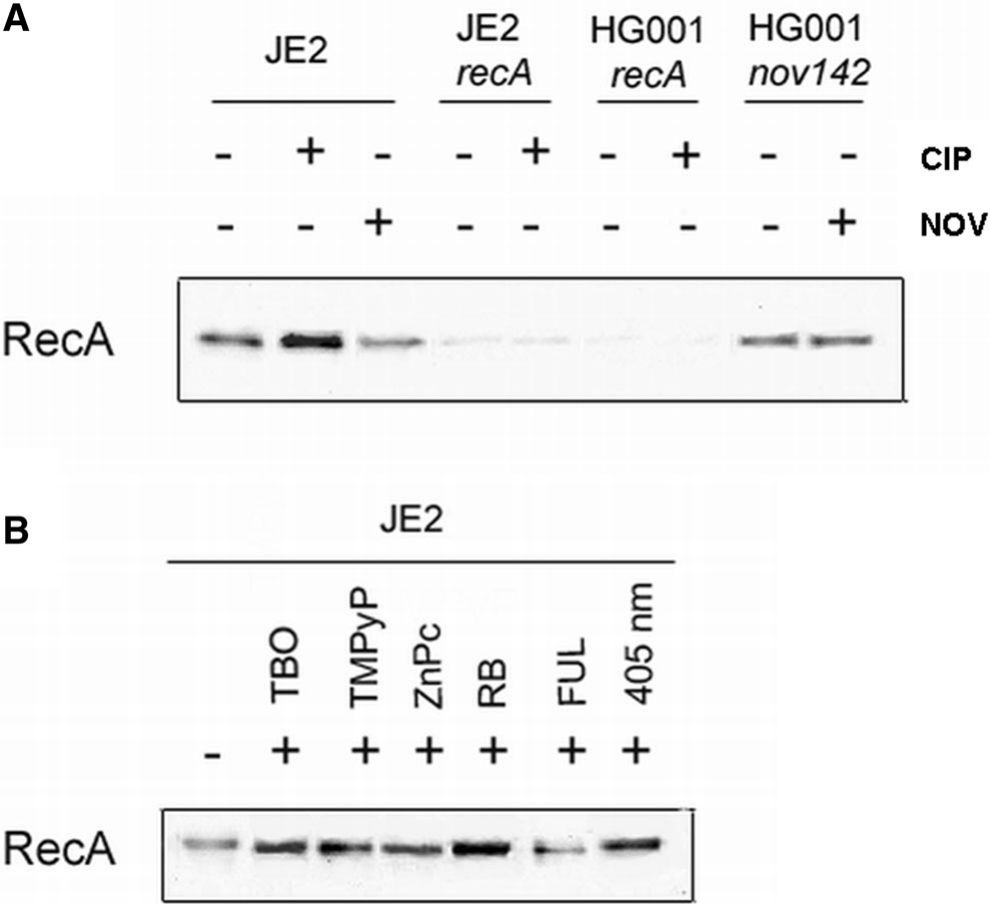
Effects of novobiocin/ciprofloxacin and phototreatments on RecA expression. The RecA protein level was detected by Western blot analysis. *S*. *aureus* strains were grown to exponential phase (OD_600_ 0.6) and treated with or without ciprofloxacin (CIP) and novobiocin (NOV) for 1 h. **a** Effects of the antibiotics on RecA level. **b** Effects of the phototreatments on the RecA level

**Fig. 7:**
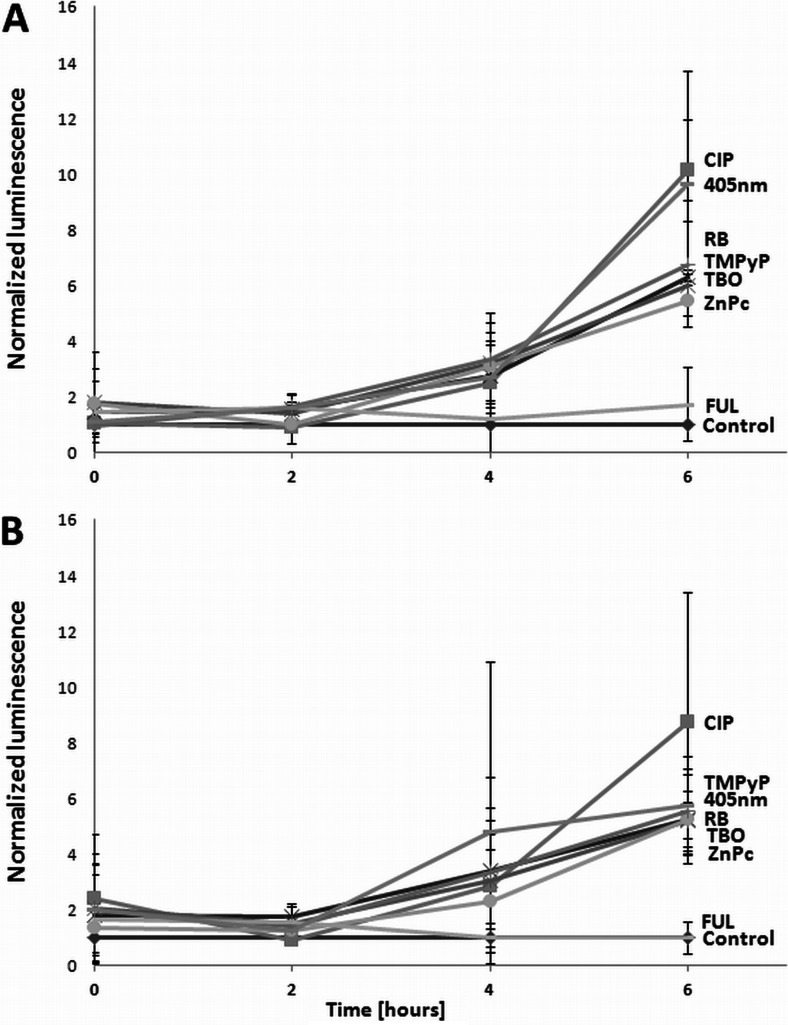
Expression of SOS genes. The luminescence of **a** *S*. *aureus* RN4220 *recA*-*lux* and **b** RN4220 *lexA*-*lux* treated with ciprofloxacin (CIP) or photoinactivating conditions in broth was measured using a Wallac 1420 Victor multilabel counter (Perkin-Elmer). The control consisted of bacteria that were maintained in the dark. The means of three separate experiments are shown, and the *bars* represent the S.D.

Because phototreatment induced DNA damage (Fig. 1), and its efficacy depended on RecA level (Figs. 3 and 4), this treatment should also result in increased *recA* expression. To analyze the effects of antibiotics (CIP and NOV) and phototreatment on RecA expression, both factors were applied to exponentially growing bacteria, and the levels of RecA protein were determined using immunoblotting of lysates from bacteria treated with or without antibiotics and/or photoinactivation. We primarily verified whether the *S*. *aureus* strains used herein expressed previously described levels of RecA and responded accordingly to the proposed mechanism of NOV and CIP. Ciprofloxacin is a well-known RecA-inducing agent and was used as a positive control for increased RecA expression (Schroder et al. 2013). The wild-type USA300 JE2 strain responded adequately to the administered antibiotics (increased RecA upon ciprofloxacin and reduced RecA upon novobiocin treatment) (Fig. 6a). Both *S*. *aureus* mutant strains containing a disruption of the *recA* gene exhibited low expression level of RecA (Fig. 6a). Finally, the strain expressing a GyrB enzyme that is not susceptible to novobiocin (HG001 *nov142*) showed no reduction of RecA in response to novobiocin treatment (Fig. 6a). We also verified that at sublethal doses of phototreatments in the presence of photosensitizer, the levels of RecA protein increased (Fig. 6b) except fulleropyrrolidine treatment. To verify that the expression of *recA* was also increased at the mRNA level, the *recA* promoter-*lux* constructs were used to enable the monitoring of increased gene expression by measuring the luminescence (Mesak et al. 2008). The starting cultures of *S*. *aureus* with an optical density at 595 nm of 0.150 were treated photodynamically or with CIP (positive control), and the luminescence of the samples was recorded every 2 h (Fig. 7a). The results of luminescence assays were in agreement with Western blot analysis. Each photosensitizer treatment, excluding fulleropyrrolidine, increased in the bioluminescence signal indicating induction of *recA* expression (Fig. 7a).

### RecA determines the response of *S*. *aureus* to phototreatment independent to LexA

Induction of ssDNA formation during phototreatment may increase RecA level. RecA interacts and promotes the self-cleavage of LexA leading to the derepression of *recA* and other SOS response genes. By using luminescence assay in *lexA*-*lux* and *recA*-*lux* strains, we showed both *lexA* and *recA* expression induced by phototreatments (Fig. 7).To investigate the role of RecA alone without interference from LexA, we used the *S*. *aureus* strain with uncleavable LexA (HG001 *lexAG94E*). We found the response of HG001 *lexAG94E* to photoinactivation was not significantly different from the wild-type strain (Fig. 5).

### Blue light (405 nm) treatment is dependent on endogenous porphyrins

**Fig. 8:**
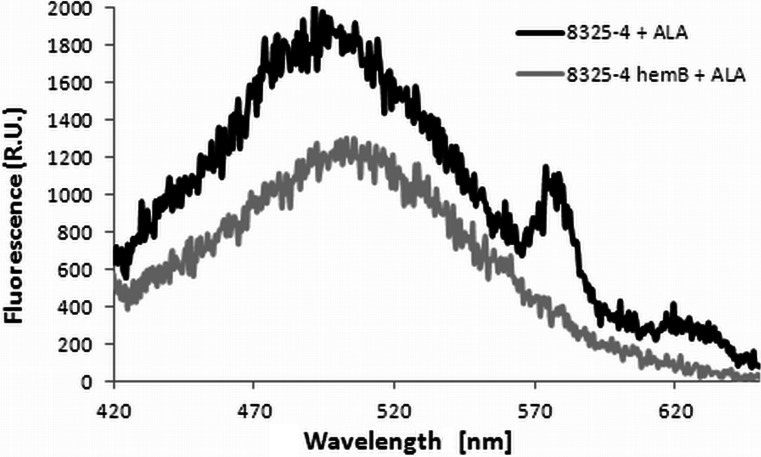
Fluorescence emission spectra of ALA-induced porphyrins in *S*. *aureus* NCTC8325-4. The bacteria were incubated with 1 mM ALA for 48 h (37 °C, 150 rpm). Next, extracellular porphyrins were extracted from the growth medium supernatant after lyophilization and sonication (10 min). After extraction of the porphyrins, their fluorescence spectra were determined using a Perkin Elmer EnVision spectrofluorometer, with an excitation wavelength of 405 nm

**Fig. 9:**
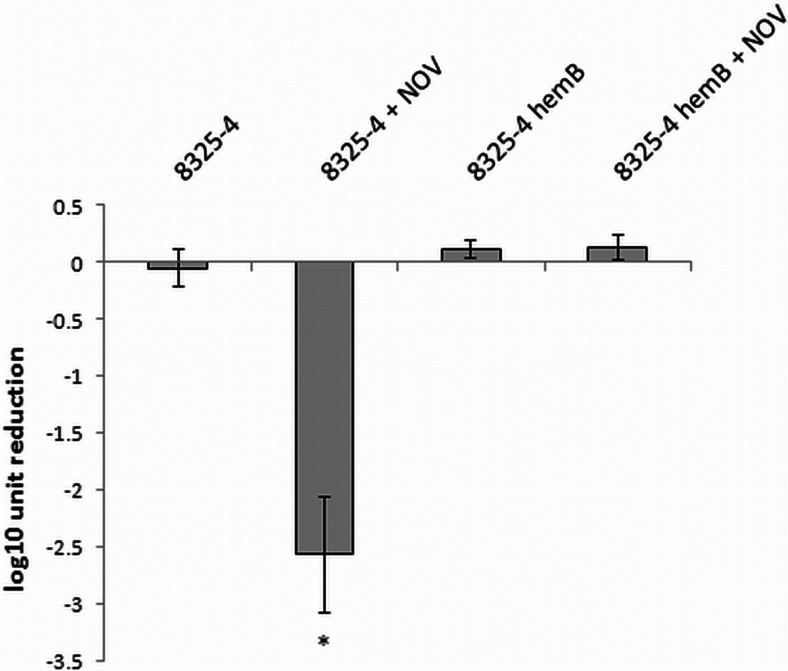
Photoinactivation efficacy against *S*. *aureus* NCTC8325-4. Microbial cell planktonic suspensions of *S*. *aureus* NCTC8325-4 and the Δ*hemB* mutant in mid-log phase were illuminated (405 nm, irradiance 5 mW/cm^2^, fluence 10 J/cm^2^). For the novobiocin-mediated experiments, mid-log phase cultures were preincubated with antibiotic (MIC) for 1 h at 37 °C. Values represent the means of three separate experiments and *bars* represent the S.D. Significance of comparisons to the control, *p < 0.05. *NOV* novobiocin

Because the major mechanistic studies in the current work were conducted with blue light (405 nm) treatment, we verified whether the underlying mechanism of the bactericidal activity of blue light (405 nm) included endogenously produced photosensitizers or other factors that are involved in its antibacterial activity. To address this question, we used the *S*. *aureus* wild-type reference strain (NCTC 8325-4), which is capable of endogenous porphyrin production, and its isogenic knockout mutant (8325-4 Δ*hemB*), displaying a stable, electron transport-deficient small colony variant (SCV) phenotype (Von et al. 1997). *hemB* is a part of the porphyrin biosynthetic pathway encoding the aminolevulinic acid dehydrase (porphobilinogen synthase) (Granick and Beale 1978). Besides slow growth, SCVs are characterized by many common features such as reduced pigmentation and metabolic changes (Kriegeskorte et al. 2014). The lack of the ability to produce endogenous porphyrins was determined spectrophotometrically by fluorescence measurements of *S*. *aureus* culture supernatants after excitation with light wavelengths of 405 nm. To induce endogenous porphyrin production, the *S*. *aureus* cultures were exposed to delta-aminolevulinic acid. As shown in Fig. 8, only the wild-type strain was able to produce endogenous porphyrins, as indicated by the specific fluorescence peaks at 580 and 630 nm. To determine whether this ability is required for the bactericidal activity of blue light (405 nm), the light treatment was applied to the *hemB* mutant and parental strain (Fig. 9). As expected, exposure of the wild-type *S*. *aureus* strain to light treatment resulted in a greater reduction of viable cells when the cells were preincubated with novobiocin. In contrast to these findings, the *hemB* mutant strain displayed no decrease in viability following irradiation, supporting the requirement for endogenously produced porphyrins to produce the bactericidal activity of the 405-nm light treatment (Fig. 9).

### Phototreatment does not contribute to the increased *S*. *aureus* mutability

**Fig. 10:**
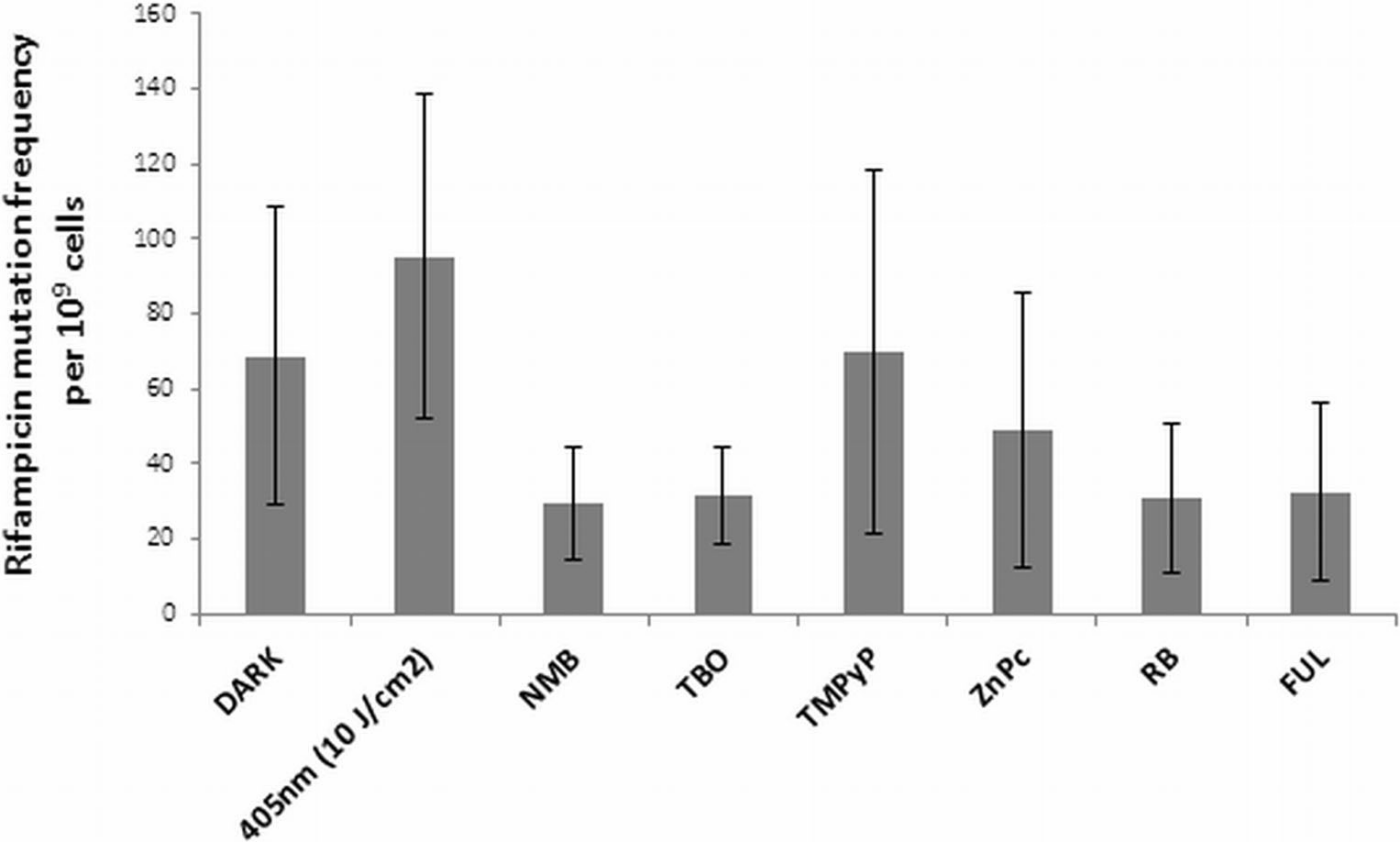
Effect of phototreatments on the development of resistance. *S*. *aureus* USA300 JE2 cultures in mid-log phase were treated photodynamically and plated on BHI plates as well as plates supplemented with rifampicin (0.25 mg/l) to determine the mutation frequency. The mutation frequency was defined as the ratio of resistant colonies in relation to the total number of bacteria (CFU). The *bars* represent the mean values of three biological replicates + S.D.

Previous studies have suggested that oxidative stress might prompt genotypic variation among bacterial species (Boles and Singh 2008). Phototreatment causes DNA damage and RecA activation, and one can assume that the SOS response could also be induced. This event leads to DNA repair and might result in an increased mutation frequency through the induction of error-prone polymerases such as Umu (Cirz et al. 2007). Because the error-prone polymerase UmuC plays a key role in accelerating the mutation frequency, we determined the influence of phototreatment on the development of spontaneous resistance to rifampicin. To investigate whether photo-induced oxidative stress might increase the mutability of *S*. *aureus*, we examined the effects on mutation frequencies in response to antibiotic treatment using a 4× MIC rifampicin concentration in culture medium. Planktonic *S*. *aureus* USA300 JE2 did not demonstrate significant differences in mutation frequency in response to phototreatment in comparison to basal mutation frequencies (Fig. 10).

### Ames mutagenicity assay

**Fig. 11:**
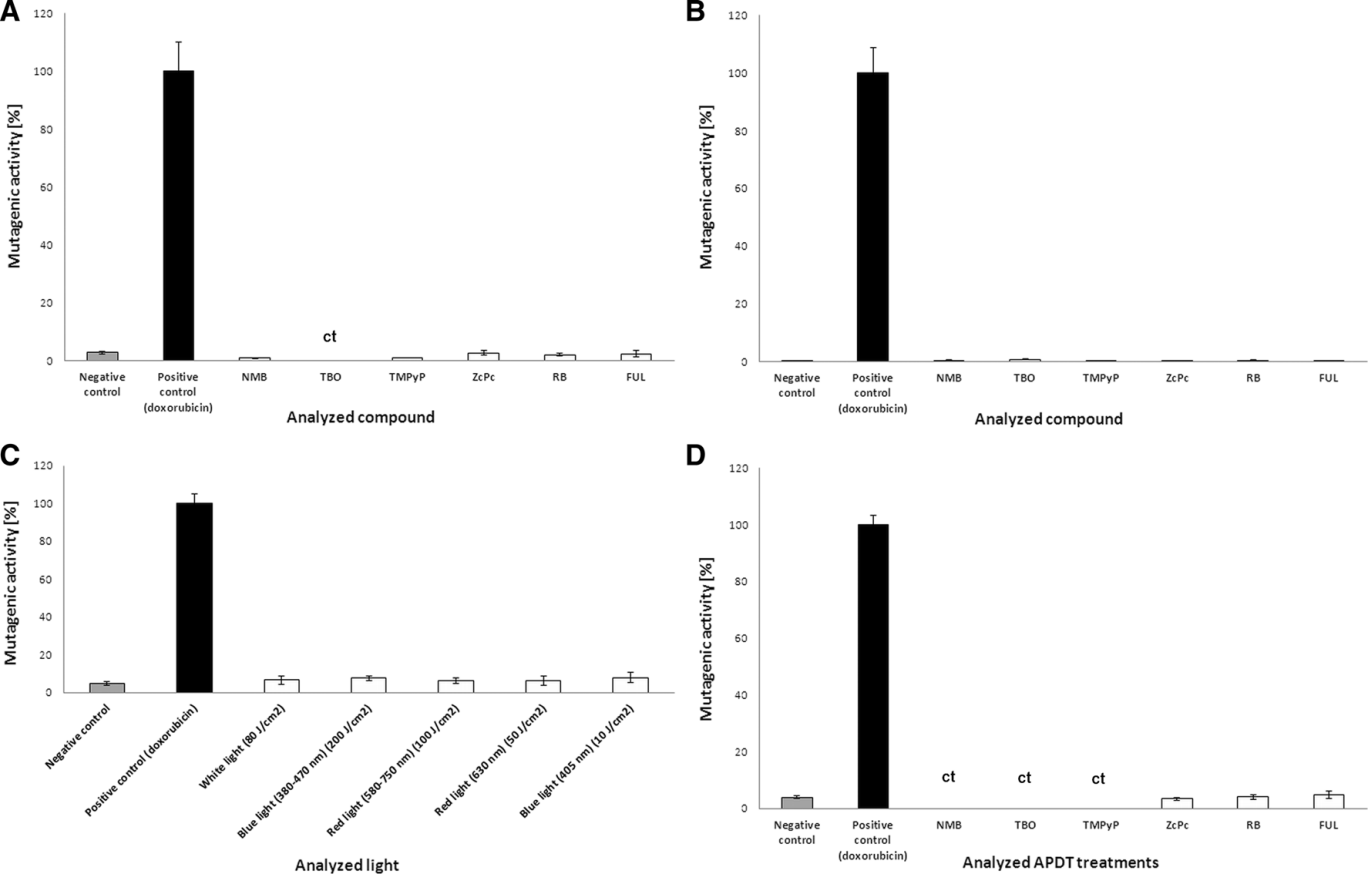
Mutagenic activity of the analyzed photosensitizers and light in the *S*. Typhimurium TA98 mutagenicity assay (Ames test). **a** Mutagenic activity of analyzed photosensitizers in Ames test without mutagenic activation. The results are reported as a percentage of the positive control (doxorubicin, 90 ng per plate; 909 ± 72 (±SD) revertants per plate) ± SD. **b** Mutagenic activity of the analyzed photosensitizers in the Ames test with mutagenic activation. The results are reported as a percentage of the positive control (2-amino-3-methylimidazo[4,5-f]quinoline, 10 μg per plate; 4887 ± 432 (±SD) revertants per plate) ± S.D. **c** Mutagenic activity of the analyzed light treatments in the Ames test without mutagenic activity. The results are reported as the percentage of the positive control (doxorubicin, 90 ng per plate; 325 ± 16 (±S.D.) revertants per plate) ± S.D. **d** Mutagenic activity of the analyzed photosensitizer-light combinations in the Ames test without mutagenic activity. The results are reported as the percentage of the positive control (doxorubicin, 90 ng per plate; 452 ± 14 (±S.D.) revertants per plate) ± S.D.; *ct* observed cytotoxic effects

We used the *S*. Typhimurium TA98 strain to investigate the mutagenic activity of photosensitizers (without light activation), light doses, and photodynamic treatment (photo-induced sensitizers), both without and with the activating microsomal S9 fraction (Fig. 11). The mutagenic effects of sensitizers that were not activated are shown in Fig. 11a, b. All of the analyzed compounds were non-mutagenic against bacterial cells in the absence and presence of the S9 fraction. The exposition of bacterial cells to light doses used for sensitizer activation revealed no mutagenic activity of the light alone (Fig. 11c). Finally, no mutagenic activity was reported for the phototreatments examined herein (sensitizers/light combinations) (Fig. 11d). However, regarding NMB, TBO, and TMPyP, the analyzed treatment conditions were cytotoxic to the *S*. Typhimurium strain, and its mutagenic activity could not be determined properly (Fig. 11d). A high level of cytotoxicity was also detected for TBO without light activation (Fig. 11a).

## Discussion

It is generally accepted that DNA damage occurs when cells are intensively photoinactivated or are no longer viable (Alves et al. 2014). Data published by George and Kishen (2008) based on a DNA damage study indicated that photoinactivation using methylene blue (MB) could cause extensive damage to the chromosomal DNA of *Enterococcus faecalis* cells when more than 3 log_10_ unit reduction in cell concentration was obtained. Because a close association between the photosensitizer and the target is required to cause the deleterious effect, the data indicated that MB molecules can penetrate the cell wall of bacteria and damaged their chromosomal DNA. However, when MB was applied in doses that only reduced less than 2 log_10_ bacterial cells, the DNA damage was considerably lower. Similar results were obtained by Caminos et al. (2008) in their studies of *Escherichia coli* photosensitization with TMPyP. Significant damage of genomic DNA has been reported in studies of photodynamic treatment’s doses that kill bacterial cells for more than 2 log_10_ unit reduction (Caminos et al. 2008). The same effect was observed for zinc phthalocyanine (ZnPc) by Spesia et al. (2009) (Spesia et al. 2009). Therefore, the role of DNA as a target in the photodynamic process that influences cell viability has been underestimated, and membrane proteins are considered to be major targets of photo-induced oxidation. In the present work, in agreement with a thesis that cell envelopes are the major targets of photoinactivation, we attempted to evaluate the actual contribution of DNA damage to the outcome of phototreatment. The results indicate that photo-induced DNA damage can occur in cells that are still viable or when sublethal treatment was applied and less than 1–2 log_10_ cell reduction was detected (Fig. 1). Moreover, we showed that DNA damage can be easily detected even without disruption of the cell membrane integrity (Fig. 2), which indicated that if the DNA repair mechanism was activated during the course of the photodynamic treatment, then the bacterium could overcome the induced DNA damage and avoid inactivation. In agreement with our previously published data, no DNA damage was reported in fulleropyrrolidine-sensitized *S*. *aureus* cells (Grinholc et al. 2015). This result was probably due to the low cell-penetrating ability of the sensitizer. In the case of fulleropyrrolidine treatment, a high level of cell envelope integrity disruption has been reported in response to activation with light, indicating that this compound binds to the cell envelope and exerts its bactericidal activity in close proximity (Grinholc et al. 2015).

For numerous bacteria, the SOS response has been recognized as a critical factor in the response to stress, in particular to induced damage to DNA, which is processed to single-stranded DNA (Cirz et al. 2007). RecA forms filaments on the single-stranded DNA, facilitating repair via recombination and stimulating the autoproteolysis of the SOS gene repressor LexA. This cleavage inactivates the LexA repressor and results in the induction of SOS genes. Many classes of antibiotics, including fluoroquinolones, e.g., ciprofloxacin, and β-lactams, have been shown to induce LexA cleavage and the SOS response via the upregulation of *recA* expression (Schroder et al. 2013; Miller et al. 2004). LexA controls the expression of *recA* and *lexA*, the positive and negative regulators of the response, as well as genes encoding proteins involved in DNA repair or recombination and the DNA polymerases required for the induced mutation (Cirz et al. 2007). In addition, in previous studies, we confirmed the strong activation of *recA* expression by ciprofloxacin in *S*. *aureus* and subsequently demonstrated that aminocoumarins (novobiocin) lead to the repression of *recA* expression (Schroder et al. 2013). In the present work, we attempted to evaluate the significance of photo-induced DNA damage by investigating the *S*. *aureus* SOS pathway in response to emergent DNA breaks. The results revealed that partial inhibition of the SOS machinery resulted specifically from the downregulation of *recA* expression or disruption of the *recA* gene, and consequently, the efficacy of the photodynamic treatment could be significantly enhanced (Figs. 3, 4, and 5). This finding suggests that photoinactivation results in severe DNA damage that leads to cell death if the DNA repair systems are inhibited. Moreover, the induction of DNA breaks in viable cells leads to increased levels of RecA protein as well as upregulation of the *recA* gene (Figs. 6 and 7). However, the observed phenomena were not reported for fulleropyrrolidine because no DNA damage was detected following the administration of this compound (Fig. 1a) (Grinholc et al. 2015). It is not likely that the increased susceptibility of *S*. *aureus* to photoinactivation resulted solely from the downregulation of *recA* expression when administered with novobiocin. Thus, the observed phenomenon was confirmed in a novobiocin-resistant *S*. *aureus* mutant strain expressing a non-susceptible GyrB enzyme (HG001 *nov142*). This strain showed no reduction of RecA levels in response to treatment with novobiocin (Fig. 6a), and as expected, no increased susceptibility to phototreatment was observed (Fig. 5). This result indicates that the effect of novobiocin on the phototreatment efficacy is mediated by antibiotic-dependent GyrB inhibition and is unlikely to be due to additional effects on other potential targets. These findings could be translated into clinical value because the use of *recA* downregulating agents could provide increased bactericidal activity during photodynamic treatment for both exogenously administered sensitizers (Fig. 3) and endogenous porphyrins (blue light treatment) (Figs. 4, 5, and 9).

The *recA* gene is preceded by a LexA-binding motif, and thus, the upregulation of *recA* in response to phototreatment presumably occurs through LexA cleavage. Thus, we utilized a previously constructed mutant in which LexA was rendered uncleavable (Schroder et al. 2013). In a previous study, we showed that the basal level of *recA* in untreated *S*. *aureus* cultures was not significantly different between wild-type and the *lexAG94E* mutant (Schroder et al. 2013). Furthermore, as expected, the addition of ciprofloxacin did not result in enhanced *recA* transcription in *lexAG94E* indicating that *recA* expression could not be upregulated in this strain (Schroder et al. 2013). The response of HG001 *lexAG94E* to photoinactivation was not significantly different when compared to the wild-type strain, which indicated that the basal level of RecA was sufficient to determine the susceptibility of *S*. *aureus* to the photo-mediated treatments (Fig. 5). Our results indicate that RecA activity, and no other LexA target genes, is needed for bacterial response to photoinactivation. This is in line with our next observation that the mutation frequencies are not altered because *umuC* is probably not expressed. This result suggests that the mechanism employed to potentiate the bactericidal efficacy of photoinactivation resides in the inhibition of *recA* prior to irradiation (i.e., in response to novobiocin pretreatment).

The mutation plays an important role in the development of antibiotic resistance in *S*. *aureus* and other staphylococci (Woodford and Ellington 2007). A single point mutation can cause clinically significant levels of resistance to a variety of antimicrobials, such as rifampicin, mupirocin, fusidic acid, and fluoroquinolones. Moreover, in cases of the accumulation of mutations at multiple loci, resistance to glycopeptides could be achieved (Kato et al. 2010). Thus, the identification of any factor that elevates the basal mutation frequency is a high priority. Consequently, investigations to determine whether the proposed treatment leads to the emergence of antibiotic resistance are a key issue. In principle, the mutagenic activity of antimicrobials is considered to occur within concentrations that are very close to the MIC because higher concentrations may have bactericidal effects and damage most of the cells in the population. In contrast, lower concentrations do not provide a stimulatory effect (Couce and Blazquez 2009). Thus, in the current work, we investigated the mutagenic effect of phototreatments at sublethal doses (reducing cell viability by <2 log_10_ units) by evaluating the appearance of mutants carrying resistance to rifampicin. Here, we showed that, although DNA damage and RecA activation could be observed, photoinactivation did not increase the mutation frequency (Fig. 10). This result is likely due to the lack of *umuC* activation, which represents induction of the late SOS response, and a phototreatment exposure duration of several minutes (in recent studies, the longest phototreatment exposure was 72 min). However, in the present study, the expression of *umuC* was not investigated, and thus, further studies are needed. We also conducted the bacterial mutagenicity test (Ames test) using one of the reference strains: *S*. Typhimurium TA98. The Ames test is the standard mutagenicity test performed prior to the introduction of new drug candidates (McCarren et al. 2011). This test is accepted by numerous international organizations and fulfills ISO guidelines (Reifferscheid et al. 2012). To be confirmed as non-mutagenic, the drug candidate must be analyzed using five reference strains—three in the *Salmonella* genus and two in the *Escherichia* genus (Mortelmans and Zeiger 2000); however, the analysis of one strain is sufficient to describe the compound as non-mutagenic and recommends further investigations, especially basic science research. Very limited published data have assessed the mutagenicity of phototreatments and related sensitizers. Thus, the present findings provide a significant improvement of our knowledge regarding photodynamic treatments. Casteel et al. (2004) performed genotoxicity assays using a non-activated sensitizer (without illumination) and showed that the revertant counts did not increase in comparison to background levels, indicating that none of the studied porphyrins was mutagenic in *S*. Typhimurium TA98 (Casteel et al. 2004). The results presented herein indicate that the photosensitizers assessed are non-mutagenic both without and with metabolic activation. It is important to note that although the analyzed compounds are applied to the skin, they may be absorbed and transferred via the blood to the liver, where they can undergo enzymatic reactions catalyzed by cytochrome P450. Therefore, assessments of the mutagenic activity using the S9 fraction allowed us to reject the concept that this activity might be exerted by metabolites of the compounds evaluated. In addition, although the *S*. Typhimurium TA98 strain is sensitive to light (especially UV light) (Mortelmans and Zeiger 2000), treatment of the samples with light did not produce any response, which would have been observed as an increased number of His^+^ revertants. Unfortunately, in the cases utilizing new methylene blue, toluidine blue O, which is also toxic when tested without light and metabolic activation, and TMPyP, the combination of light and photosensitizer was toxic to the bacterial strain assessed (Fig. 11a, d). However, the main purpose of therapy is to inhibit bacterial growth, and thus, these effects might be attributed to the efficiency of the proposed combinations in killing bacterial strains together with an increased susceptibility to external factors. Nevertheless, to fully confirm the non-mutagenic and non-toxic properties of the analyzed photosensitizer-light combinations, they should be tested both in other bacterial strains recommended for the Ames test and eukaryotic cell lines. Finally, one must be conscious that the absence of an increased number of rifampicin-resistant *S*. *aureus* mutants and of mutagenic activity toward *S*. Typhimurium may also have resulted from the phototreatment conditions and duration of exposure used in our experiments. These variables may not have been capable of selecting for rifampicin-resistant variants or His^+^ revertants, and further studies examining a range of concentrations as well as a prolonged duration of exposure should be considered.

**Fig. 12:**
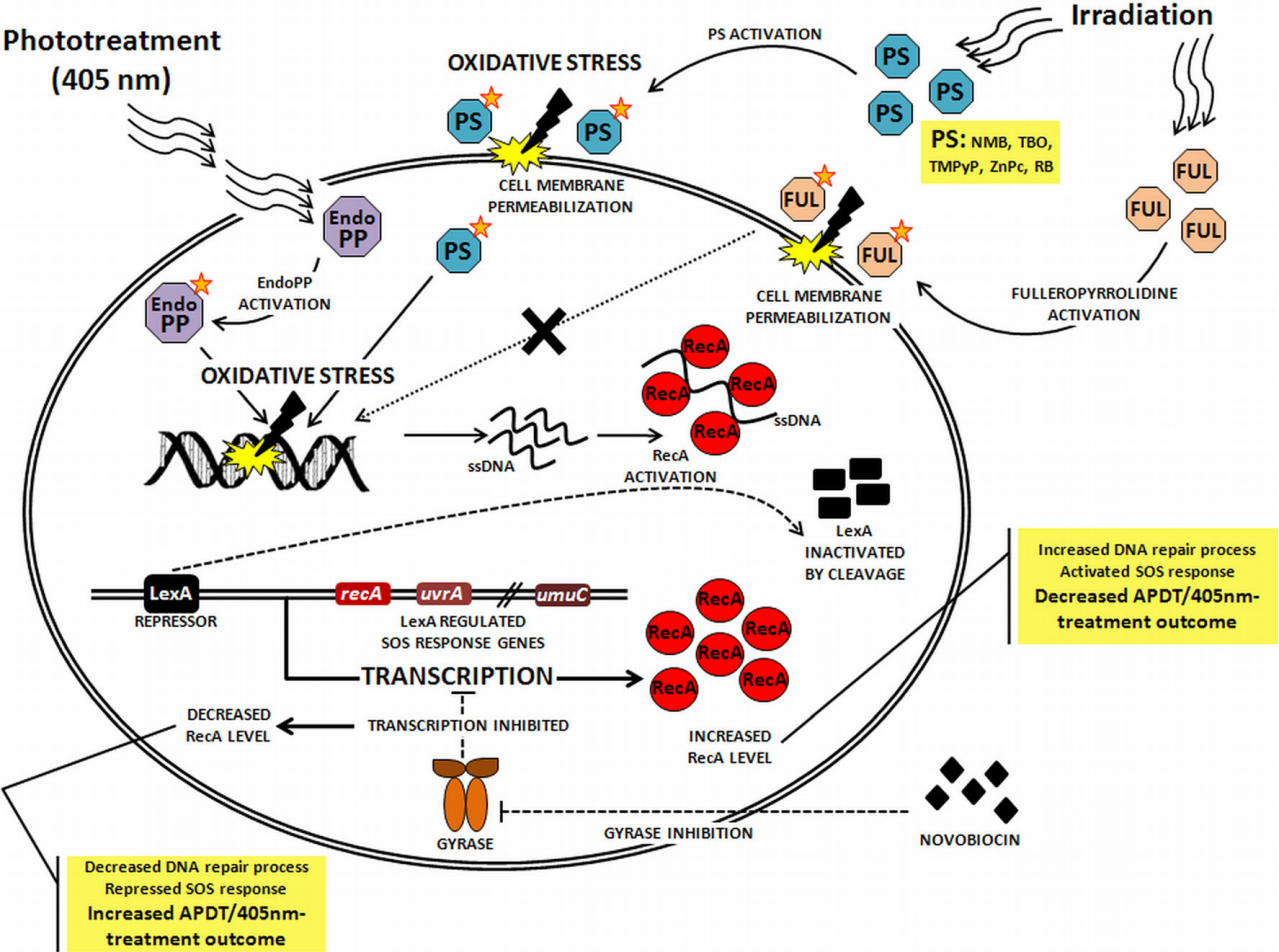
Proposed mechanism underlying the photoinactivation process. Irradiated sensitizer molecules achieve an activated state and lead to the production of reactive oxygen species as well as free radicals (oxidative stress). This process results in cell membrane as well as DNA damage (excluding the poor-cell-penetrating fulleropyrrolidine). The SOS machinery is operated by two key regulators: the SOS repressor LexA and the inducer RecA. RecA responds to DNA damage by binding to ssDNA, which triggers autocleavage of LexA. The LexA repressor dissociates from the SOS boxes and induces transcription of the SOS regulon. Novobiocin binds to the GyrB subunit of gyrase and inhibits transcription, leading to the decrease in *recA* expression. *APDT* antimicrobial photodynamic therapy, *PS* photosensitizer, *EndoPP* endogenous porphyrins, *NMB* new methylene blue, *TBO* toluidine blue O, *TMPyP* 5,10,15,20-tetrakis(1-methyl-4-pyridinio)porphyrin tetra(*p*-toluenesulfonate), *ZnPc* zinc phthalocyanine, *RB* Rose Bengal, *FUL* fulleropyrrolidine

In the present study, we aimed to investigate the mechanistic features of the photoinactivation process. Our findings are summarized in the Fig. 12, which shows the proposed mechanism of photoinactivation involving the SOS machinery. Moreover, the results of the present study, in comparison to previously published data, provide strong evidence that the inactivation of bacteria using 405-nm light results from the photostimulation of endogenous porphyrins. This study provides proof-of-concept evidence that DNA damage is the significant factor triggered by the photoinactivation process and also occurs in phototreated but viable bacterial cells. If these cells are not treated with agents that affect DNA repair mechanisms, then they would achieve a harmless state and would not be inactivated. Thus, we explored the effect of RecA inhibition on the efficacy of phototreatment. Our results using the *recA*-defective strain and the application of a *recA*-downregulating agent (novobiocin) suggest that the effectiveness of photoinactivation depends in part on RecA activity, thus supporting the hypothesis that RecA inhibition is a potential therapeutic adjuvant in combination with photodynamic approaches.
